# Christmas 1912 at Cambridge

**Published:** 1913-01-04

**Authors:** 


### CHRISTMAS 1912 AT CAMBRIDGE.

### How it was Prepared and How it was Spent.

Preparation for Christmas festivities at Addenbrooke's Hospital, Cambridge, began many weeks ago, every member of the staff taking part each day in his or her share—some engaged in making decorations, others organising entertainments, nurses devoting their little evening leisure to practising as a Pierrot troupe, and such like. By December 24 all the decorations, or material for them, had been obtained. On this day the tremendous task of bedecking every ward, corridor, etc., throughout the hospital commenced quite early, and by mid-day all was aglow. A wonderful transformation scene was the result. At 8 p.m. on Christmas Eve there assemble in the out-patients' waiting-hall the Vicar of St. Mary-the-Great, Cambridge, with the organist and members of the choir, the chaplain of the hospital, the matron, sisters, and nurses. The lights of the hospital are for the most part turned down, and this great company, each member of which carries a lighted Japanese lantern, wends its way singing carols in each and every ward.

On Christmas Day every patient received a parcel containing useful articles of clothing. At twelve o'clock noon Christmas dinner was served in each ward, where the special carvers, in fancy dress, were this year the House Physician, Dr. Gerald Walker; the House Surgeon, Dr. H. D. Barnes; the second House Surgeon, Dr. Gordon W. Spencer; Dr. Anson Jordan; and Mr. Humphry Jordan. At 1.30 the domestic staff had their Christmas dinner of turkey, plum-pudding, etc., dessert—being waited upon by the matron, sisters, and doctors.

The Christmas tree was in the Victoria (Women's Surgical) Ward and electrically illuminated. The tree was about 15 feet in height, and loaded to such an extent that every patient and every member of the entire hospital staff received a gift off it. At 5 p.m. Father Christmas arrived in a motor-car, and accompanied by a fairy and the elephant and monkey, with their attendant or keeper, being heralded in by a brass and drum band.

At 8 p.m. the same day the man-servants and maid-servants enjoyed their usual Christmas party, for on Tuesday, December 31, the season's festivities were gradually brought to a close by a tea in Hope Ward. As far as possible patients were in each case brought from other wards, so that they had in many cases the privilege of enjoying six entertainments.

